# Serogroups, subtypes and virulence factors of shiga toxin-producing *Escherichia coli* isolated from human, calves and goats in Kerman, Iran

**Published:** 2018

**Authors:** Rohollah Taghadosi, Mohammad Reza Shakibaie, Hesam Alizade, Hossein Hosseini-Nave, Asma Askari, Reza Ghanbarpour

**Affiliations:** 1 *Department of Microbiology and Virology, Kerman University of Medical Sciences, Kerman, Iran*; 2 *Infectious and Tropical Disease Research Center, Hormozgan Health Institute, Hormozgan University of Medical Sciences, Bandar Abbas, Iran*; 3 *Department of Pathobiology, Faculty of Veterinary Medicine, Shahid Bahonar University of Kerman, Kerman, Iran *

**Keywords:** Shiga toxin-producing E. coli, serogroup, virulence factors

## Abstract

**Aim:**

The present study was conducted to detect the occurrence, serogroups, virulence genes and phylogenetic relationship of shiga toxin-producing *Escherichia coli* (STEC) in human, clave and goat in Kerman (southeast of Iran).

**Background:**

STEC have emerged as the important foodborne zoonotic pathogens causing human gastrointestinal disease and confirming the risk to public health.

**Methods:**

A total of 671 fecal samples were collected from diarrheic patients (n=395) and healthy calves (n=156) and goats (n=120) and screened for the presence of *stx* gene. Furthermore, the prevalence of *stx1* and *stx2* variants, serotypes (O157, O145, O103, O26, O111, O91, O128, and O45), phylogenetic groups and the presence of *ehxA*, *eae*, *hylA*, *iha* and *saa* virulence genes were studied.

**Results:**

Prevalence of STEC in human diarrheic isolates was 1.3% (5 isolates), in claves was 26.3% (41 isolates) and in goats was 27.5% (33 isolates). *stx1* gene was the most prevalent variant and detected in 75 isolates. Furthermore, *stx1c* was the most predominant *stx* subtype, found in 56 isolates. The *ehxA* identified in 36 (45.6%) isolates, followed by *iha* 5 (6.3%), *eaeA* 4 (5.1%), *hlyA* 2 (2.5%) and *saa* 2 (2.5%). Most of the isolates belonged to phylogroup B1. Only two O26 and one O91 isolates were detected in our study.

**Conclusion:**

Our results show that STEC strains were widespread among healthy domestic animals in the southeast of Iran

## Introduction

 Shiga toxin-producing by *Escherichia coli* (STEC) is an important enteric pathogen, has been reported in several outbreaks with clinical manifestations including mild diarrhea, hemorrhagic colitis (HC) and hemolytic uremic syndrome (HUS) (1, 2). The disease in human is primarily a food-borne infection. Although STEC strains have been isolated from other animals such as goats, sheep, swine, wild animals and humans, cattles are the major source of food contamination (3). The ability of STEC strains to cause human disease is mainly due to the production of shiga-like toxins (stx) which are classified into two closely related subgroups, stx1 and stx2 (encoded by the *stx1* and *stx2* genes). Stx1 is a homologous group with only three variants (*stx1a*, *stx1c*, and *stx1d*), while *stx2* is a more heterogeneous group and is comprised of several subtypes (*stx2a*, *stx2b*, *stx2c*, *stx2d*, *stx2e*, *stx2f* and *stx2g*) (4, 5). STEC strains producing *stx2a*, *stx2c*, or *stx2d* subtypes are more associated with HC and HUS in humans. In contrast, *stx2b*, *stx2e*, *stx2f* and *stx2g* are related to animal infections (6). Additional factors that contribute to virulence have also been described, including intimin (encoded by the eae gene), involved in the attachment of *E. coli* to the enterocyte, plasmid-encoded enterohemolysin (encoded by *ehxA* gene) which acts as a pore-forming cytolysin, alpha-hemolysin (encoded by the *hlyA* gene), IrgA homologue adhesin (*iha*) which is a STEC adherence-conferring molecule and Saa which is an autoagglutinating adhesin produced by LEE-negative STEC (3, 7-9). Epidemiologic investigations demonstrated that O157 is the main cause of HC and HUS in human; however, additional serogroups that have been reported in human clinical cases are O26, O45, O91, O103, O111, O128 and O145, and others in recent years (10, 11). 

*E. coli* can also be assigned to one of the four major phylogenetic groups (A, B1, B2 and D) based on the presence or absence of *chuA*, *yjaA* and TspE4.C2 (12). 

Bearing in mind the importance of *E. coli* as food-borne pathogens, as vehicle of human disease, the objectives of this study were to investigate the distribution of subtypes, serotypes, virulence factors and phylogenetic groups among STEC strains from healthy domestic animals (calves and goats) and patients with diarrhea in Kerman, southeast of Iran. 

## Methods

**Specimen collection and microbiological processing**

In a prospective study, from October 2014 to November 2015, a total of 671 fecal samples were collected from diarrheic patients (n=395) and fecal healthy calves (n=156) and goats (n=120). The human samples were related to both male (n=215) and female (n=180). Their age ranged from <5 years old (n=107), 5 to 15 years old (n=146), 15 to 40 years old (n=75) and 40 to 90 years old (n=67). The human isolates obtained from the rectal swab of the patient with diarrhea referred to Afzalipour and Payambar-Azam hospitals. All animal samples were collected by veterinarians from School of Veterinary Medicine, Shahid Bahonar University, Kerman, Iran. All samples were placed into Amies medium (Becton Dickinson, BBL, and USA) and were sent out to the laboratory in ice-cooled containers. The samples were taken to the microbiology laboratory, Kerman University of Medical Sciences and identified as *E. coli* by biochemical characteristics and conventional diagnostic tests (13). All strains were stored at −70°C in Trypticase Soy broth (Difco Laboratories, Detroit, Mich.) containing 30% glycerol for further study.

**Detection of STEC strains**

For the detection of *stx* gene, DNA template was obtained by boiling method (14). Presence of *stx *gene in the selected *E. coli* colonies was verified by PCR method (15). In addition, *stx*-positive isolates were examined for the presence of *stx*_1_ and *stx*_2_ genes by using duplex-PCR (16). A positive control for PCR was *E. coli* Sakai (*stx*_1_+/*stx*_2_+/*eaeA*+). The *E. coli* strain MG1655 was used as a negative control for virulence genes. Details of the primers and the length of the expected amplification product are listed in Table 1.

**Identification of subtype genes**

We used PCR method for determination of *stx*_1_ and *stx*_2_ subtypes. PCR for detection of *stx*_1a_, *stx*_1c_, *stx*_1d_, *stx*_2a_, *stx*_2b_, *stx*_2c_, *stx*_2d_, *stx*_2e_, *stx*_2f_ and *stx*_2g_ subtypes was carried out by methods described previously (17-19) (Table 1).

**Identification of serogroup genes**

Furthermore, PCR assay was used for the identification of O157, O145, O103, O26, O111, O91, O128 and O45 as described by Hemmatinezhad *et al.* (20) (Table 1).

**Identification of virulence genes**

The presence of following virulence genes *ehxA*, *eae*, *hylA*, *iha* and *saa* were detected by PCR assay (21-24) (Table 1).

**Determination of STEC strains phylogenetic groups**

Strains assigned to one of the four main phylogenetic group of *E. coli* (A, B1, B2 and D) by using a PCR-based method as described previously (12). The genomic DNA of bacterial strains amplified by triplex-PCR using primers targeted at three markers, *chuA*, *yjaA* and TspE4.C2.

**Table 1.** Oligonucleotide Primers Used in this Study

References	Annealing temp (°C)	Size (bp)	Primer sequence (5’-3’)	Target gene
15	55	518	GAGCGAAATAATTTATATGTG TGATGATGGCAATTCAGTAT	*stx*
16	58	180	TAAATCGCCATTCGTTGACTAC AGAACGCCCACTGAGATCATC	*stx*_1_
16	60	255	GGCACTGTCTGAAACTGCTCC TCGCCAGTTATCTGACATTCTG	*stx*_2_
18	57	219	CACGTTACAGCGTGTTGCA CGCCCACTGAGATCATCC	*stx*_1a_
17	54	498	TTTTCACATGTTACCTTTCCT CATAGAAGGAAACTCATTAGG	*stx*_1c_
18	57	192	CTTTTCAGTTAATGCGATTGCT AACCCCATGATATCGACTGC	*stx*_1d_
18	55	969	AGATATCGACCCCTCTTGAA GTCAACCTTCACTGTAAATG	*stx*_2a_
19	60	251	AAATATGAAGAAGATATTTGTAGCGGC CAGCAAATCCTGAACCTGACG	*stx*_2b_
17	55	124	GCGGTTTTATTTGCATTAGT AGTACTCTTTTCCGGCCACT	*stx*_2c_
17	58	175	GGTAAAATTGAGTTCTCTAAGTAT CAGCAAATCCTGAACCTGACG	*stx*_2d_
17	56	267	ATGAAGAAGATGTTTATAGCG TCAGTTAAACTTCACCTGGGC	*stx*_2e_
19	60	424	TGGGCGTCATTCACTGGTTG TAATGGCCGCCCTGTCTCC	*stx*_2f_
19	62	573	CACCGGGTAGTTATATTTCTGTGGATATC GATGGCAATTCAGAATAACCGCT	*stx*_2g_
12	59	279	GACGAACCAACGGTCAGGAT TGCCGCCAGTACCAAAGACA	*chu*A
12	59	211	TGAAGTGTCAGGAGACGCTG ATGGAGAATGCGTTCCTCAAC	*Yja*A
12	59	152	CTGGCG AAAGACTGTATCAT CGCGCCAACAAAGTATTA CG	TspE4.C2
24	61	1551	GGTGCAGCAGAAAAAGTTGTAG TCTCGCCTGATAGTGTTTGGTA	*ehx*A
21	61	1177	AACAAGGATAAGCACTGTTCTGGCT ACCATATAAGCGGTCATTCCCGTCA	*hylA*
23	57	119	CGTGATGAACAGGCTATTGC ATGGACATGCCTGTGGCAAC	*saa*
23	57	827	CTGGCGGAGGCTCTGAGATCA TCCTTAAGCTCCCGCGGCTGA	*iha*
22	65	1087	CAGGTCGTCGTGTCTGCTAAA TCAGCGTGGTTGGATCAACCT	*eae*A
20	58	259	CGGACATCCATGTGATATGG TTGCCTATGTACAGCTAATCC	O157
20	58	609	CCATCAACAGATTTAGGAGTG TTTCTACCGCGAATCTATC	O145
20	58	406	TAGAGAAATTATCAAGTTAGTTCC ATAGTTATGAACATCTTGTTTAGC	O111
20	58	291	GCTGACCTTCATGATCTGTTGA TAATTTAACCCGTAGAATCGCTGC	O91
20	58	289	GCTTTCTGCCGATATTTGGC CCGACGGACTGATGCCGGTGATT	O128
20	58	527	CCGGGTTTCGATTTGTGAAGGTTG CACAACAGCCACTACTAGGCAGAA	O45
20	58	321	TTGGAGCGTTAACTGGACCT GCTCCCGAGCACGTATAAG	O103
20	58	423	CAGAATGGTTATGCTACTGT CTTACATTTGTTTTCGGCATC	O26

**Statistical analysis**

SPSS version 15.0 software for Windows (SPSS Inc., Chicago) was used for statistical analysis. *P* values of less than 0.05 were considered to be significant. 

## Results

Among 671 *E. coli* isolates isolated from healthy farm calves, goats and patients with sign of diarrhea, 79 strains were positive for the presence of *stx* gene and identified as STEC. Among STEC strains 41 strains were positive in claves, 33 strains in goats and 5 strains in human samples. Our results showed that 54 (68.4%) of the strains carried *stx*_1_ only, 4 (5.1%) contained *stx*_2_ only, and 21 (26.6%) possessed both *stx*_1 _and *stx*_2_.

Two *stx*_1_ subtypes (*stx*_1a_ and s*tx*_1c_) and four *stx*_2_ subtypes (*stx*_2a_, *stx*_2b_, *stx*_2c _and *stx*_2d_) were detected with a total of 13 different *stx*_1_ and *stx*_2_ subtypes combinations as shown in Table 2. Among the subtypes, *stx*_1c_ was detected in 56 strains, followed by *stx*_1a_ (25 strains), *stx*_2b_ (20 strains), *stx*_2c_ (19 strains) and *stx*_2a_ (1 strain). In addition, *stx*_2d_ (11 strains) was detected only in combination with other *stx* genes (Table 2). There was no correlation between *stx* subtypes and animal sources (*P*≤0.05).

**Table 2:** Summary of *stx* subtyping in 79 non O157-STEC strains isolated from calves, goats and human fecal samples

No (%) of strains	source			*stx* subtype					
	humans	calves	goats	*stx*_1a_	*stx*_1c_	*stx*_2a_	*stx*_2b_	*stx*_2c_	*stx*_2d_
35 (44.3)	2	20	13	-	+	-	-	-	-
16 (20.3)	1	6	9	+	-	-	-	-	-
8 (10.1)	0	4	4	-	+	-	+	+	+
5 (6.3)	0	5	0	-	+	-	+	+	-
5 (6.3)	0	2	3	+	+	-	-	-	-
2 (2.5)	2	0	0	-	-	-	+	-	-
2 (2.5)	0	2	0	+	+	-	+	+	+
1 (1.3)	0	0	1	+	-	-	-	+	-
1 (1.3)	0	1	0	-	+	-	+	-	-
1 (1.3)	0	0	1	+	-	+	-	-	-
1 (1.3)	0	0	1	-	-	-	+	+	-
1 (1.3)	0	1	0	-	-	-	+	+	+
1 (1.3)	0	0	1	-	-	-	-	+	-
Total= 79	5	41	33	25	56	1	20	19	11

No = Number of non O157-STEC strains, % = percent of non O157-STEC strains; ‒ = negative, + = positive.

**Table 3:** Distribution of STEC in phylogenetic groups from calves, goats and human fecal samples

Phylogenetic group	No. (%) of STEC strains isolated from			Total
	humans	calves	goats	
A	0 (0)	6 (14.6)	6 (18.2)	12 (15.2)
B1	0 (0)	35 (85.4)	27 (81.8)	62 (78.5)
B2	0 (0)	0 (0)	0 (0)	0 (0)
D	5 (100)	0 (0)	0 (0)	5 (6.3)

NO = Number of STEC strains isolated from calves, goats and human fecal samples, % = percent of STEC strains isolated from calves, goats and human fecal samples

The STEC strains were further tested for five putative virulence factors, including *eae*A, *hly*A, *iha*, *ehx*A and *saa*. Out of 79 strains, 49 (62%) carried at least one virulence gene tested. The *ehx*A was detected in 36 (45.6%), *eae*A in 4 (5.1%), *iha* in 5 (6.3%), *hly*A in 2 (2.5%) and *saa* in 2 (2.5%) of the isolates.

Phylogroup B1 was the most prevalent (62/79; 78.5%) among the STEC strains, followed by phylogroups A (12/79; 15.2%) and D (5/79; 6.3%). As shown in Table 3, all isolates of human origin belonged to the D Phylogroup. In this study, phylogenetic group B2 was not detected in STEC strains.

Serogroup analysis showed that none of the isolates belonged to O45, O103, O111, O128, O145 and O157 serogroups, while O26 and O91 were detected in two (clave and goat) and one (clave) isolate, respectively.

## Discussion

STEC can be found in various food sources, transmission of this pathotype from undercooked or unpasteurized animal products to human is problematic (1, 10). It is estimated that STEC to cause more than 265,000 illnesses each year in the USA, with more than 3,600 hospitalizations and 30 deaths (25). In the present study, STEC strains were isolated just from 1.2% of patients with diarrhea which was consistent with previous studies (26-28). According to a survey, high variability of genes-encoding *stx* was detected in the *E. coli* isolates in HIV and thalassemia patients in Kerman, south-east of Iran. Among *E. coli* isolates from faecal samples, 30.8% isolates were positive for *stx* genes (34). However, 26.8% of *E. coli* isolated from goats and calves carried at least one of the *stx* genes. This frequency was, lower compared to reports from Spain and Brazil (37% and 44%) (29, 30), but higher from results reported in Iran (8.5%) (31). Another study from West Azerbaijan province in Iran revealed that 21.92% of the *E. coli* isolates recovered from fecal healthy calves harbored *stx* genes (32). These variations may likely be due to geographical and climatic conditions and differences in the natural intestinal flora present in animal’s gastrointestinal tract (33).

In STEC strains characterized in this study, *stx*_1_ was the most common *stx* gene identified, a result which is similar to previous reports (31, 34). In contrast, some studies have detected *stx*_2_ as a dominant *stx* gene in fecal samples of animals (35, 36). Although, this variant mainly found in strains isolated from healthy human carriers and most likely does not cause severe diseases in human (36).

In the present study, *stx*_1c_ was the predominant variant among the STEC strains isolated. *Stx*_1c_ Subtype has also frequently been reported in previous studies (5, 37). However, *stx1c*-encoding strains are associated with asymptomatic human carriage or mild illness (38). *Stx*_2c_ and *stx*_2d_ are associated with HUS. However, they are less toxic on Vero cells compared to *stx*_2a_. STEC strains with *stx*_2a _are associated with several clinical symptoms, such as HUS and HC (39). Stephan and Hoelzle suggested that *stx*_2b_ was not associated with severe human diseases, because most strains carrying *stx*_2b_ were isolated from healthy human carriers (40). In the present study, two strains carrying only *stx*_2b_ were isolated from human and it was possible that these two STEC strains were not the main causative agent of diarrhea. In this study, two strains isolated from calves carried 5 subtypes of *stx*_1a_, *stx*_1c_, *stx*_2b_, *stx*_2c_, *stx*_2d_ simultaneously. The combination of five *stx* genes in one isolate had not been previously reported. In the study of Bertin *et al.* strains with a combination of *stx*_1_ and/or *stx*_2_ subtypes were found to be more toxic toward Vero cells than other strains (41). In our study, other *stx*_2_ subtypes such as *stx*_2e_, *stx*_2f_ and *stx*_2g_ were not found. These subtypes are related to animal infections (42). 

In addition, we studied the distribution of eight important serotypes in the above isolates which associated more frequently with HUS and HC. None of the isolates belonged to O45, O103, O111, O128, O145 and O157 serogroups, while O26 and O91 were detected in two and one isolates respectively. This finding is in agreement with the failure to find these serotypes in yaks and cattle (8, 43). It seems that in some regions, ruminants are not important reservoirs for the outbreak isolates. Although, human infections with stx-producing *E. coli* O26 is uncommon and has resulted in less severe illness, but is a major cause of HUS in Europe continent (44).

In this study, only four strains contained *eae*A gene; however, none of the isolates carried the *stx*_2_ subtypes. The low frequency of the *eae*A gene found in the present study may be related to the low frequency of certain serogroups, as it has been reported that the presence of the *eae*A gene is associated with specific O serogroups of STEC, such as the O157, O145, O103, O26, and O111 (33). Since the majority of the STEC strains lacked *eae*A gene, we investigated other factors associated with adherence including *iha* and *saa*. These two virulence factors have been reported to be highly important for pathogenicity of *eae*-negative STEC strains (8). Only 2.5% and 6.35% of strains were positive for* saa *and *iha *respectively. It is possible that other virulence factors, that were not investigated in the present study like *lpfa* and *paa* play important role in the adherence of STEC strains. Also, we detected *ehx*A and *hly*A genes in 45.6% and 2.5% of strains respectively. Overall, the frequency of virulence factors in STEC isolates was lower than that observed in other studies (8, 45). Carriage of *stx* gene positive *E. coli* isolates in the gastrointestinal tract of healthy ruminants proposes that these are transient commensal bacteria in these animals and the virulence genes of these isolates were either not or very poorly expressed (32).

Investigation on STEC phylogroups indicated that majority of commensal and diarrhogenic strains are belonged to group B1 and A, while extra intestinal *E. coli* strains belong mainly to group B2 and D (46). In this study, phylogenetic group B2 were not detected in STEC isolates, which was consistent to previous study (46). However, like in many studies, phylogenetic group B1 was predominant among isolates from animals (47, 48). All of the human strains belonged to phylogenetic group D2, while it was not found in strains isolated from animals. 

In conclusion, although STEC strains were widespread among healthy domestic animals in the southeast of Iran, prevalence of STEC in patient with diarrhea was low and most of the STEC strains did not belong to O serogroups that are commonly associated with severe disease in humans. Furthermore, these strains were mainly belonged to phylogenetic group B1. These facts together with the high prevalence of *stx*_1c_, *stx*_2b_, *stx*_2c_ subtypes and low prevalence of *stx*_2a_, suggest that most of STEC in Iranian calves and goats may not pose a serious public health concern.
